# Exploring parental perspectives on the barriers and benefits of pediatric cochlear implantation: a cross-sectional study in Jeddah, Saudi Arabia

**DOI:** 10.3389/fped.2026.1795907

**Published:** 2026-04-15

**Authors:** Aya M. Baageel, Shahad M. Aldini, Tuleen J. Abudawood, Hanin H. Rayes

**Affiliations:** Department of Speech-Language Pathology and Audiology, Faculty of Medical Rehabilitation Sciences, King Abdulaziz University, Jeddah, Saudi Arabia

**Keywords:** cochlear implantation, hearing loss, pediatric, rehabilitation, quality of life, parental barriers, Saudi Arabia

## Abstract

**Background:**

Parents of children with severe-to-profound hearing loss face various challenges that can hinder access to timely interventions. Previous literature has studied various factors affecting parental experiences with cochlear implantation, but research remains limited in Saudi Arabia. Therefore, this study aimed to investigate the challenges faced by parents of children who are candidates for cochlear implantation surgery or have already undergone the procedure and to assess children's quality of life (QoL) after cochlear implantation.

**Method:**

A cross-sectional study was conducted among 123 parents of children aged 9 months to 18 years in Saudi Arabia, including cochlear implant (CI) recipients (*n* = 104) and candidates (*n* = 19). Participants were recruited from CI clinics in Jeddah and from online platforms across the country. Data were collected using two questionnaires: a structured tool titled Parental Barriers to Pediatric Cochlear Implantation, which was reviewed by an expert panel to assess perceived barriers, and the validated Arabic version of the 24-item Glasgow Children's Benefit Inventory (GCBI) to evaluate post-implantation QoL. Descriptive and inferential statistics were utilized.

**Results:**

The results revealed that most parents expressed concerns about the surgical procedure (60.1%), including potential risks and outcomes. A substantial proportion reported concerns regarding post-implantation expenses such as the cost of device maintenance (49.6% strongly agreed) and rehabilitation sessions (48.8% strongly agreed). Additionally, 69.1% of the parents reported difficulties accessing rehabilitation services due to the lack of rehabilitation centers in their area. The GCBI questionnaire showed positive outcomes for children's overall QoL (88.1%). Significant improvements were observed in the functional, developmental, cognitive, and social-emotional domains of children's QoL, rather than in the health or medical need domains.

**Conclusion:**

Key parental barriers to pediatric cochlear implantation include financial concerns, limited access to rehabilitation, and post-surgical challenges. Despite this, parents reported positive outcomes, particularly in the functional, cognitive, and social-emotional domains.

## Introduction

According to the World Health Organization (1), the number of children experiencing disabling hearing loss is greater than 7.5 million worldwide. The estimated prevalence of disabling hearing loss in the Middle East and North Africa is 3.17% (1). In Saudi Arabia, a study conducted by Al-Shaikh and Zakzouk (2) revealed that 0.72% of 10,000 children were diagnosed with severe-to-profound sensorineural hearing loss. Despite the significant increase in reported hearing loss cases globally, there is a notable lack of recent studies examining the prevalence of severe to profound sensorineural hearing loss among children in Saudi Arabia.

Children born with or developing severe to profound hearing loss often require cochlear implantation. Prior to implantation, the child typically undergoes a comprehensive audiological assessment, along with experience in home-based interventions and a trial of hearing aids, to ensure that they have met the cochlear implant (CI) candidacy criteria (3). According to Alzahrani et al.'s guidelines for cochlear implantation in Saudi Arabia (4), candidates are children aged 9 months and older with bilateral severe to profound sensorineural hearing loss exceeding 70 dB HL, who show no benefit after a 3–6-month hearing aid trial with ongoing assessment of speech, language, and auditory skills. Additionally, the criteria require no contraindications such as the absence of the auditory nerve.

Early implantation is essential for avoiding delays in language acquisition during the critical period of language development. Implantation before the age of two years has been proven to yield promising results in terms of speech perception and intelligibility by providing timely access to auditory input (5, 6). In fact, a study by Dettman et al. (7) reported that 81% of 403 children who received CIs before 12 months of age exhibited normal vocabulary development. While early implantation has been shown to yield significant benefits, studies have found that the average age for cochlear implantation still exceeds two years, which is considered late for optimal outcomes (6, 8). These findings highlight the importance of early detection, supporting the implementation of Saudi Arabia's mandatory national newborn hearing screening (NBHS) program that was launched in 2016 by the Ministry of Health (9). Although this NBHS program was mandated, implementation has varied across regions, particularly during the early phases of program expansion. Coverage may differ depending on place of birth, healthcare sector, and referral pathways, which may contribute to delayed identification in some cases.

Several factors can contribute to the observed delay, one of which is the challenges faced by parents, hindering their ability to proceed with surgery (10). Despite the established safety and efficacy of CI surgical procedure, which often is completed within 1.5–3 h for unilateral CI cases and up to 4.5 h for bilateral cases (11, 12), delays in proceeding with surgery remain common. Dev et al. (13) identified that parents often have concerns about the surgical procedure and potential complications during or after the procedure, making them uncertain about the best course of action for their child (13).

Other factors that impede the pursuit cochlear implantation include lack of awareness, parental fears, medical comorbid conditions of the child, financial difficulties of the family, variability in referral pathways, logistical constraints, and lack of available healthcare services in the area of residence (10). Additionally, parental hesitation can also stem from personal experiences of stigma, depending on whether or not the parents also have hearing loss, in addition to social acceptance and cultural beliefs within the community (e.g., deaf communities) (14).

Co-morbid medical conditions can also contribute to the cochlear implantation delays among pediatric candidates (15, 16). In fact, a study by Birman et al. (17) revealed that comorbid conditions such as developmental delays, syndromic diagnoses, and neurological disorders were prevalent among CI candidates, affecting approximately one-third of the study's participants (*n* = 88). These conditions often delay implantation because of the need for additional assessments and potential surgical risks. The results of the study also indicate that children with comorbid conditions may experience less improvement in communication outcomes after implantation. Similarly (16), reported that exclusive oral communication and stronger cognitive abilities emerged as significant predictors of better language performance for children with IC. Collectively, these findings might affect the parent's decision regarding the procedure.

Expenses related to the cochlear implantation surgery, CI device maintenance, and post-implantation rehabilitation can be overwhelming for parents experiencing financial constraints (18, 19). Armstrong et al. (15) demonstrated that children without insurance or those covered by public insurance were more likely to receive cochlear implantation at a later age than those with private insurance. In Saudi Arabia, however, cochlear implantation is primarily provided through government-funded healthcare services, reducing direct financial burden for eligible citizens. Nonetheless, regional disparities, indirect costs (e.g., travel and time away from work), and differences in service distribution may still affect timely access to rehabilitation. Limited availability of services in rural areas compared to urban centers further contributes to access challenges (20).

Another factor influencing the decision-making process is parents' expectations and confidence in their children's potential outcomes of CIs (18). A systematic review by Warner-Czyz et al. (21) substantiates this by demonstrating that CIs significantly enhance children's Quality of Life (QoL), as assessed through a detailed questionnaire, particularly in the domains of communication and social relationships. Consistent with these findings, Yu et al. (22) reported significant longitudinal improvements in children's QoL following cochlear implantation, with trajectories varying across centers and influenced by factors such as age at implantation, service-delivery and device configuration. Moreover, a study by Fan et al. (23) reported a notable improvement in children's QoL when they were implanted at a younger age, received optimal auditory input, underwent speech rehabilitation before implantation, and used implants over an extended period. Furthermore, studies have emphasized the importance of NBHS for early detection of congenital hearing loss to facilitate timely intervention, thus leading to better communication outcomes (15, 19).

International research has extensively examined parental barriers to cochlear implantation and consistently demonstrated significant improvements in children's QoL following implantation. Longitudinal data further confirm sustained QoL gains, with outcomes influenced by contextual and service-related factors. These findings suggest that while QoL benefits are well established globally, their magnitude and determinants may vary across healthcare systems and cultural settings. In Saudi Arabia, evidence on this topic remains limited. Given the distinct healthcare system, early intervention pathways, and sociocultural context in Saudi Arabia, population-specific investigation is needed. Therefore, this study examined parental barriers to cochlear implantation and evaluated its impact on children's QoL within the Saudi context. We hypothesize that parents, in Saudi Arabia, who are considering CI surgery for their children face various barriers that impede or delay the cochlear implantation process and its accessibility. Additionally, we hypothesized that parents of children who received CI would perceive significant communication benefits that enhance their QoL after implantation.

## Methodology

### Study design

This study employed a cross-sectional design that utilized convenience sampling to facilitate broader participation. It was conducted over a seven-month period from October 2024 to May 2025.

### Participants

The target sample size was approximately 246 participants, calculated based on an initial pilot study which identified the prevalence of parents expressing concerns about cochlear implantation to be 65%, with a 90% confidence level, and 5% margin of error. The inclusion criteria were parents of children aged 9 months to 18 years living in Saudi Arabia whose children were either CI candidates eligible for surgery or recipients who had already undergone the procedure. The exclusion criteria included parents of children with contraindications (e.g., congenital cochlear malformation and absence of the auditory nerve) to minimize anatomical heterogeneity, as inner ear anomalies may independently influence surgical complexity, auditory performance, and language outcomes, potentially confounding the variables under investigation. Furthermore, individuals not residing in Saudi Arabia were also excluded to reduce contextual heterogeneity. Participants were recruited from CI clinics in Jeddah and online platforms across Saudi Arabia. A total of 123 parents participated in this study.

### Outcomes, exposures, and data collection

The primary outcomes were parent-reported barriers to pediatric cochlear implantation and QoL following implantation. Parental barriers were assessed using the Parental Barriers to Pediatric Cochlear Implantation Questionnaire, which captures concerns related to surgical risks, financial burden, access to rehabilitation services, psychosocial factors, and decision-making challenges. This questionnaire was developed independently; however, certain questions were adapted from a previously published study (18) and reviewed by an expert panel consisting of an audio-vestibular physician, an audiologist, and a speech language pathologist, and the common feedback provided was incorporated into the questionnaire.

Child QoL outcomes were evaluated among cochlear implant recipients using the validated Arabic version of the 24-item Glasgow Children's Benefit Inventory (GCBI) (24) covering functional, developmental, cognitive, social-emotional, and medical domains. Parents of children who had undergone surgery were asked to complete both questionnaires consecutively, while parents of children who had not yet undergone surgery were only asked to complete the first questionnaire. Completion of the GCBI questionnaire was optional, as it required additional time from participants. Of the 104 cochlear implant recipients, 101 completed the GCBI. Three responses were excluded due to incomplete data.

The primary exposure was cochlear implantation status (recipient vs. candidate), while secondary exposures included the child's demographic and clinical characteristics, parental sociodemographic factors, and family level variables such as income, nationality, and consanguinity.

### Confounders and sources of bias

Potential confounders included socioeconomic status, access to healthcare services, and clinical timing factors (e.g., age at diagnosis and implantation) given their plausible associations with both exposures and outcomes. Potential sources of bias included selection bias due to convenience sampling and regional over-representation, recall, and information bias arising from the parent-reported questionnaires. Clear, structured, and cross-check questions (e.g., hearing devices) were included to verify responses.

### Ethical considerations

This study was approved by the Institutional Ethics Review Board of the King Abdulaziz University Hospital [Reference no. (15–25)]. Parents of the children participating in the study were provided with a digital information sheet outlining the study's purpose, procedures, and potential benefits. The information sheet was followed by an informed consent statement confirming parents’ willingness to participate in the study. The participants were able to withdraw at any stage while completing the questionnaire until submission. Parents of the CI recipients were given the option to either proceed to the second questionnaire or submit only the first. All responses were collected anonymously and stored securely in a storage system (Google Drive) accessible only to researchers. The participants had the opportunity to receive a virtual audiological consultation session from the research team. No physical or psychological risks were expected as participation was limited to online questionnaires. This study adhered to the ethical guidelines of the Declaration of Helsinki.

### Statistical plan

Descriptive statistics were used to summarize the demographic data, barriers, and benefits with averages and percentages to provide an overview of the common challenges and perceived advantages. Data were analyzed using the Statistical Package for the Social Sciences (SPSS) software (Version 22.0; IBM Corp., Armonk, NY). The chi-squared goodness-of-fit (*χ*^2^ GoF) test was used to examine the deviation of the sample from an equal distribution. The chi-squared test of independence was used to examine the relationships between categorical variables. Cramér's *V*-test was used to examine the strength of the association between the variables. Fisher's exact test was used to examine the significance of the relationships between variables when the chi-square test was not applicable or when its assumptions were violated.

## Results

### Children's demographic information

A total of 123 participants, comprising both CI recipients (84.6%) and CI candidates (15.4%), were included in the study. Most were Saudi nationals (82.1%), with over half residing in the Makkah region (54.5%). In terms of gender, the sample was relatively balanced (53.7% male and 46.3% female). Most children were aged 6–12 years (34.1%). More than half of the children were attending day care or school (64.2%). A considerable number of children (42.3%) did not undergo NBHS. Correspondingly, delayed identification was evident, with over half of the patients being diagnosed at or after 9 months of age (56.1%). The results also revealed that almost all children (99.2%) had bilateral hearing loss, whereas only 0.8% of the children had unilateral hearing loss. Regarding the age at implantation, the majority of the children received CIs between the ages of 2 and 3 years (46.2%), and less than a quarter were implanted before the age of 2 years (26.9%). Among CI recipients, 62.5% of the children were implanted bilaterally, while 37.5% of the children received unilateral implantation. Refer to Table 1 for the children's demographic and clinical summary.

**Table 1:** Demographic and clinical characteristics of children included in the study.

Section	Category	*N*	%	*Χ*^2^ GoF
Child Age	<2 years	10	8.1%	21.19***
	2–3 years	23	18.7%	
	4–5 years	23	18.7%	
	**6–12 years**	**42**	**34** **.** **1%**	
	≥13 years	25	20.3%	
Gender	Male	66	53.7%	0.66
	Female	57	46.3%	
Daycare/School	No	44	35.8%	9.96**
	**Yes**	**79**	**64** **.** **2%**	
Age of Diagnosis	<9 months	54	43.9%	1.83
	**≥9 months**	**69**	**56** **.** **1%**	
Age of Implantation	<2 years	28	22.8%	30.54***
	**2–3 years**	**48**	**39** **.** **0%**	
	4–5 years	17	13.8%	
	≥6 years	11	8.9%	
NBHS	Fail	40	32.5%	5.42
	Pass	31	25.2%	
	Not tested	52	42.3%	
Hearing Loss	**Bilateral**	**122**	**99** **.** **2%**	119.03***
	Unilateral	1	0.8%	
Hearing Aid Use	None	46	37.4%	61.88***
	Right only	13	10.6%	
	**Both ears**	**58**	**47** **.** **2%**	
	Left only	6	4.9%	
Cochlear Implant Use	No CI	19	15.4%	58.74***
	**CI**	**104**	**84** **.** **6%**	
Implanted Ear	Right	27	22.0%	54.53***
	Left	12	9.8%	
	**Both**	**65**	**52** **.** **8%**	
Rehabilitation	No	20	16.3%	56.01***
	**Yes**	**83**	**67** **.** **5%**	
Medication Use	**No**	**118**	**95** **.** **9%**	103.81***
	Yes	5	4.1%	
Nationality	**Saudi**	**101**	**82** **.** **1%**	50.74***
	Non-Saudi	22	17.9%	
Region	**Makkah**	**67**	**54** **.** **5%**	374.07***
	AlMadina	7	5.7%	
	Riyadh	19	15.4%	
	Eastern Province	12	9.8%	
	Al-Qassim	4	3.3%	
	Tabuk	3	2.4%	
	Al-Baha	4	3.3%	
	Assir	1	0.8%	
	Hael	1	0.8%	
	Northern Borders Province	1	0.8%	
	Jazan	2	1.6%	
	Al-Jouf	2	1.6%	

Bold values represent the most frequently selected option of questions with statistically significant responses (*p* < 0.05 or lower).

** Very statistically significant (*p* < .01).

*** Extremely statistically significant (*p* < .001).

### Parental/caregiver demographic information

Parental demographics showed that most fathers (80.5%) were aged 30–49 years, most mothers (48.8%) were aged 30–39 years, and the majority of parents held bachelor's degrees. The income distribution showed that most families earned between 5,000 and 9,999 Saudi Riyal (SAR) monthly (32.5%), while only a small fraction reported earning 30,000 SAR or more (3.3%). Additionally, a large percentage of parents reported consanguineous marriage (70.7%). Refer to Table 2 for the parents' demographic and clinical summary.

**Table 2:** Sociodemographic and clinical characteristics of parents of children included in the study.

Section	Category	*n*	%	*Χ*^2^ GoF
Father's Age	<20	4	3.3%	87.04***
	20–29	5	4.1%	
	30–39	49	39.8%	
	**40–49**	**50**	**40** **.** **7%**	
	50	15	12.2%	
Mother's Age	20–29	20	16.3%	54.85***
	**30–39**	**60**	**48** **.** **8%**	
	40–49	38	30.9%	
	50	5	4.1%	
Father's Education	N/A	2	1.6%	132.73***
	Elementary	6	4.9%	
	High School	33	26.8%	
	**Bachelor**	**71**	**57** **.** **7%**	
	Postgraduate	11	8.9%	
Mother's Education	N/A	5	4.1%	125.90***
	Elementary	11	8.9%	
	High School	27	22.0%	
	**Bachelor**	**72**	**58** **.** **5%**	
	Postgraduate	8	6.5%	
Family Income	<5 k	23	18.7%	48.88***
	**5–9.9 k**	**40**	**32** **.** **5%**	
	10–14.9 k	31	25.2%	
	15–19.9 k	19	15.4%	
	20–29.9 k	6	4.9%	
	≥30 k	4	3.3%	
Consanguinity	No	36	29.3%	21.15***
	**Yes**	**87**	**70.7%**	

Bold values represent the most frequently selected option of questions with statistically significant responses (*p* < 0.05 or lower).

*** Extremely statistically significant (*p* < .001).

### Parental barriers to pediatric cochlear implantation

The participants' responses to the “Parental Barriers to Pediatric Cochlear Implantation” questionnaire revealed several significant parental barriers to cochlear implantation (Table 3). The results showed that a significant majority of parents agreed (45.5%) that they had concerns about surgical procedures, risks, and outcomes while an additional 14.6% were extremely concerned. A notable percentage of parents also agreed (35.0%) that they had difficulties accessing rehabilitation services because of the lack of rehabilitation centers in their area. Almost half of the parents also agreed that post-implantation expenses, such as the cost of device maintenance (49.6%) and rehabilitation sessions (48.8%), pose a significant challenge. Additionally, most agreed that they had concerns regarding the limitations associated with their child's ability to undergo magnetic resonance imaging (MRI) (38.2%) and participation in sports activities (41.5%).

**Table 3:** Parental barriers to pediatric cochlear implantation.

Question	Response	Frequency *n* = 123	Percent	*Χ*^2^ GoF
**Q1 Surgical Guidance:** 1. We lack adequate support from the cochlear implant team, in terms of receiving clear information and guidance about the surgical procedure, risks, and outcomes.	Strongly disagree	43	35.0%	50.82***
	**Disagree**	**56**	**45.5%**	
	Agree	18	14.6%	
	Strongly agree	6	4.9%	
**Q2 Rehab Guidance:** 2. We lack adequate support from the cochlear implant team, regarding clear information and guidance about the rehabilitation plan and expected outcomes.	Strongly disagree	38	30.9%	33.59***
	**Disagree**	**53**	**43.1%**	
	Agree	21	17.1%	
	Strongly agree	11	8.9%	
**Q3 Team Expertise:** 3. We are concerned about the expertise and experience of the medical team.	Strongly disagree	47	38.2%	39.70***
	**Disagree**	**48**	**39.0%**	
	Agree	21	17.1%	
	Strongly agree	7	5.7%	
**Q4 Surgical Risks:** 4. We are concerned about the risks and complications related to the surgery.	Strongly disagree	18	14.6%	31.31***
	Disagree	31	25.2%	
	**Agree**	**56**	**45.5%**	
	Strongly agree	18	14.6%	
**Q5 Transport Distance:** 5. We are concerned about the distance and transportation for appointments.	Strongly disagree	25	20.3%	1.46
	Disagree	32	26.0%	
	Agree	33	26.8%	
	Strongly agree	33	26.8%	
**Q6 Rehab Access** 6. We are concerned about accessing rehabilitation services due to lack of availability of rehabilitation centers in our area.	Strongly disagree	16	12.2%	18.56***
	Disagree	22	17.9%	
	**Agree**	**43**	**35.0%**	
	Strongly agree	42	34.1%	
**Q7 Surgery Cost:** 7. We find it difficult to cover the costs associated with the surgery and cochlear implant device.	Strongly disagree	33	26.8%	5.16
	Disagree	36	29.3%	
	Agree	20	16.3%	
	Strongly agree	34	27.6%	
**Q8 Post Costs:** 8. We are concerned about post-implantation expenses, such as device maintenance and repair, which present a financial challenge for us.	Strongly disagree	7	5.7%	53.16***
	Disagree	8	6.5%	
	Agree	47	38.2%	
	**Strongly agree**	**61**	**49.6%**	
**Q9 Post Rehab Costs:** 9. We are concerned about post-implantation expenses, such as rehabilitation sessions, which present a financial challenge for us.	Strongly disagree	7	5.7%	73.52***
	Disagree	18	14.6%	
	Agree	38	30.9%	
	**Strongly agree**	**60**	**48.8%**	
**Q10 Gene Therapy:** 10. We are concerned that cochlear implants may limit our child's access to medical treatments such as gene therapy.	Strongly disagree	19	15.4%	24.81***
	**Disagree**	**54**	**43.9%**	
	Agree	28	22.8%	
	Strongly agree	22	17.9%	
**Q11 MRI Limitation:** 11. We are concerned that cochlear implants may limit our child's ability to undergo MRI scans.	Strongly disagree	14	11.4%	18.30***
	Disagree	34	27.6%	
	**Agree**	**47**	**38.2%**	
	Strongly agree	28	22.8%	
**Q12 Sports Restriction:** 12. We are concerned that cochlear implants may restrict our child's participation in sports activities.	Strongly disagree	17	13.8%	21.49***
	Disagree	23	18.7%	
	**Agree**	**51**	**41.5%**	
	Strongly agree	32	26.0%	
**Q13 Social Stigma:** 13. We are concerned that cochlear implants may limit our child's interactions due to social stigma.	Strongly disagree	22	17.9%	9.33*
	**Disagree**	**44**	**35.8%**	
	Agree	32	26.0%	
	Strongly agree	25	20.3%	
**Q14 Sign Preference:** 14. We are hesitant about proceeding with cochlear implantation as we prefer to use sign language rather than relying on spoken language.	**Strongly disagree**	**101**	**80.5%**	214.00***
	Disagree	7	4.9%	
	Agree	7	5.7%	
	Strongly agree	8	6.5%	
**Q15 Family Support:** 15. We lack moral support from our family members.	**Strongly disagree**	**48**	**39.0%**	34.04***
	Disagree	45	36.6%	
	Agree	20	16.3%	
	Strongly agree	10	8.1%	
**Q16 Decision Difficulty:** 16. We find it challenging to make this decision on behalf of our child.	Strongly disagree	36	29.3%	12.19**
	**Disagree**	**44**	**35.8%**	
	Agree	21	17.1%	
	Strongly agree	22	17.9%	
**Q17 Medical Barriers:** 17. My child has other medical conditions that hinder the ability to proceed with the surgery (anatomical abnormalities).	**Strongly disagree**	**69**	**56.1%**	97.00***
	Disagree	44	35.8%	
	Agree	8	6.5%	
	Strongly agree	2	1.6%	

**Bold** values represent the most frequently selected option of questions with statistically significant responses (*p* < 0.05 or lower).

* Statistically significant (*p* < .05).

** Very statistically significant (*p* *<* .01).

*** Extremely statistically significant (*p* < .001).

#### Association between parental barriers to pediatric cochlear implantation questionnaire and implantation status

A chi-squared test of independence was conducted to examine the associations between parental barriers to the pediatric cochlear implantation questionnaire and CI recipients and candidates. The results revealed a significant association between implantation status and perceptions of team expertise [*χ*^2^ (3, *n* = 123) = 12.56, *p* = .006], with a moderate effect size (Cramér's V = .32). Parents of CI candidates were significantly more likely to report 'strongly agree' about having concerns regarding the medical team's expertise than parents of CI recipients. A strong association between implantation status and sign language preference was also evident [*χ*^2^(3, *n* = 123) = 51.61, *p* < .001], with a large effect size (Cramér's V = .648). Parents of CI recipients were significantly more likely to “strongly disagree” that sign language is a preferred alternative to CIs than parents of CI candidates. Additionally, the results revealed a strong association between implantation status and perceptions of family support [*χ*^2^(3, *n* = 123) = 15.30, *p* = .002], with a moderate effect size (Cramér's V = .353). Parents of CI recipients were significantly more likely to report “strongly disagree” or “disagree” regarding a lack of family support compared to CI candidates. Table 4 for the association between parental barriers to pediatric cochlear implantation and implantation status.

**Table 4:** Association between CI recipients and candidates and variables of parental barriers to pediatric cochlear implantation.

	Q3 Team Expertise 3. We are concerned about the expertise and experience of the medical team.				
CI	Strongly Disagree	Disagree	Agree	Strongly Agree	Chi Square (Association)
No	6 (31.6%)	4 (21.1%)	5 (26.3%)	4 (21.1%)	12.56**
Yes	41 (39.4%)	44 (42.3%)	16 (15.4%)	3 (2.9%)	
	Q8 Post Costs: 8. We are concerned about post-implantation expenses, such as device maintenance and repair, which present a financial challenge for us.				
CI	Strongly Disagree	Disagree	Agree	Strongly Agree	Chi Square (Association)
No	0 (0.0%)	4 (21.1%)	10 (52.6%)	5 (26.3%)	12.27**
Yes	7 (6.7%)	4 (3.8%)	37 (35.6%)	56 (53.8%)	
	Q14 Sign Preference: 14. We are hesitant about proceeding with cochlear implantation as we prefer to use sign language rather than relying on spoken language.				
CI	Strongly Disagree	Disagree	Agree	Strongly Agree	Chi Square (Association)
No	5 (26.3%)	6 (31.6%)	4 (21.1%)	4 (21.1%)	51.61***
Yes	96 (92.3%)	1 (1.0%)	3 (2.9%)	4 (3.8%)	
	Q15 Family Support: 15. We lack moral support from our family members.				
CI	Strongly Disagree	Disagree	Agree	Strongly Agree	Chi Square (Association)
No	2 (10.5%)	6 (31.6%)	7 (36.8%)	4 (21.1%)	15.30**
Yes	46 (44.2%)	39 (37.5%)	13 (12.5%)	6 (5.8%)	

** Very statistically significant (*p* *<* .01).

*** Extremely statistically significant (*p* < .001).

A chi-square test of independence was conducted to examine the relationship between nationality and parent-reported difficulty in surgical cost and device coverage. The chi-squared test was statistically significant, as the significant majority of non-Saudi parents agreed (36.4%) or strongly agreed (59.1%) that it was difficult to cover the costs associated with the surgery and CI device compared to Saudi parents [Q7: *χ*^2^ (*n* = 123, 3) = 29.03, *p* < .001]. The effect size measured by Cramér's V = 0.486 indicated a strong association, suggesting that nationality may be meaningfully associated with surgical and device costs. Table 5 for the association between parental barriers to pediatric cochlear implantation and nationality.

**Table 5:** Association between parental barriers to pediatric cochlear implantation and nationality.

	Q7 Surgery Cost: 7. We find it difficult to cover the costs associated with the surgery and cochlear implant device.				
Nationality	Strongly disagree	Disagree	Agree	Strongly Agree	Chi Square (Association)
Non-Saudi	0 (0.0%)	1 (4.5%)	8 (36.4%)	13 (59.1%)	29.03***
Saudi	33 (32.7%)	35 (34.7%)	12 (11.9%)	21 (20.8%)	

*** Extremely statistically significant (*p* < .001).

A chi-squared test of independence was conducted to examine the relationship between family income and parental barriers to the pediatric cochlear implantation questionnaire. The results revealed a significant association between family income and parent-reported adequacy of surgical guidance [Q1: *χ*^2^ (*n* = 123, 15) = 36.98, *p* < 0.001]. Families earning 10,000–14,900 SAR/month were significantly more likely to report “strongly disagree” regarding having difficulty finding adequate support and guidance from the CI team. However, there was a significant association between family income and parent-reported rehabilitation access [Q6: *χ*^2^ (*n* = 123, 15) = 26.70, *p* = 0.031]. Families earning ≥20,000 SAR/month were significantly more likely to report “strongly disagree” that they had concerns about accessing rehabilitation services because of the lack of availability of centers in their area. Furthermore, an association between family income and concerns about postsurgical costs, including device maintenance and repair, was evident [Q8: *χ*^2^ (*n* = 123, 15) = 27.58, *p* = .024]. For instance, families earning 20,000 SAR/month or more were significantly more likely to report “strongly disagree” that post-implantation expenses were burdensome compared to families earning <5,000 SAR/month. Table 6 for the association between parental barriers to pediatric cochlear implantation and family income.

**Table 6:** Association between parental barriers to pediatric cochlear implantation and family income.

	Q1 Surgical Guidance: 1. We lack adequate support from the cochlear implant team, in terms of receiving clear information and guidance about the surgical procedure, risks, and outcomes.				
Family Income	Strongly Disagree	Disagree	Agree	Strongly Agree	Chi Square (Association)
Less than 5 k	5 (21.7%)	10 (43.5%)	7 (30.4%)	1 (4.3%)	36.98**
5 to 9.9 k	11 (27.5%)	22 (55.0%)	5 (12.5%)	2 (5.0%)	
10 to 14.9 k	17 (54.8%)	12 (38.7%)	2 (6.5%)	0 (0.0%)	
15 to 19.9 k	8 (42.1%)	7 (36.8%)	4 (21.1%)	0 (0.0%)	
20 to 29.9 k	1 (16.7%)	4 (66.7%)	0 (0.0%)	1 (16.7%)	
20 k or more	1 (25.0%)	1 (25.0%)	0 (0.0%)	2 (50.0%)	
	Q6 Rehab Access: 6. We are concerned about accessing rehabilitation services due to lack of availability of rehabilitation centers in our area.				
Family Income	Strongly Disagree	Disagree	Agree	Strongly Agree	Chi Square (Association)
Less than 5 k	1 (4.3%)	10 (43.5%)	7 (30.4%)	1 (4.3%)	26.70*
5 to 9.9 k	2 (5.0%)	7 (17.5%)	16 (40.0%)	15 (37.5%)	
10 to 14.9 k	5 (16.1%)	4 (12.9%)	11 (35.5%)	11 (35.5%)	
15 to 19.9 k	4 (21.1%)	6 (31.6%)	3 (15.8%)	6 (31.6%)	
20 to 29.9 k	1 (16.7%)	0 (0.0%)	2 (33.3%)	3 (50.0%)	
20 k or more	3 (75.0%)	0 (0.0%)	0 (0.0%)	1 (25.0%)	
	Q8 Post Costs: 8. We are concerned about post-implantation expenses, such as device maintenance and repair, which present a financial challenge for us.				
Family Income	Strongly Disagree	Disagree	Agree	Strongly Agree	Chi Square (Association)
Less than 5 k	1 (4.3%)	1 (4.3%)	6 (26.1%)	15 (65.2)	27.58*
5 to 9.9 k	1 (2.5%)	2 (5.0%)	16 (40.0%)	21 (52.5%)	
10 to 14.9 k	2 (6.5%)	1 (3.2%)	15 (48.4%)	13 (41.9%)	
15 to 19.9 k	0 (0.0%)	3 (15.8%)	6 (31.6%)	10 (52.6%)	
20 to 29.9 k	1 (16.7%)	1 (16.7%)	2 (33.3%)	2 (33.3%)	
20 k or more	2 (50.0%)	0 (0.0%)	2 (50.0%)	0 (0.0%)	

* Statistically significant (*p* < .05).

** Very statistically significant (*p* *<* .01).

A chi-square test of independence indicated a significant association between mothers' education and parental barriers to the pediatric cochlear implantation questionnaire. Mothers with postgraduate education were significantly more likely to report that they “strongly agree” that they had concerns regarding the adequacy of surgical guidance [*χ*^2^ (12, *n* = 123) = 25.32, *p* = .013]. Moreover, a significant association between mothers' education and the perceived burden of post-implantation costs was demonstrated [*χ*^2^ (12, *n* = 123) = 26.92, *p* = .008]. For example, mothers with a postgraduate education were significantly more likely to “strongly disagree” with post-implant costs. Refer to Tables 6, 7.

**Table 7:** Association between parental barriers to pediatric cochlear implantation and mother's education.

Mothers' Education	Q1 Surgical Guidance 1. We lack adequate support from the cochlear implant team, in terms of receiving clear information and guidance about the surgical procedure, risks, and outcomes				
	Strongly Disagree	Disagree	Agree	Strongly Agree	Chi Square (Association)
N/A	2 (40.0%)	2 (40.0%)	1 (20.0%)	0 (0.0%)	25.32*
Elementary	2 (18.2%)	6 (54.5%)	3 (27.3%)	0 (0.0%)	
High School	10 (37.0%)	12 (44.4%)	5 (18.5%)	0 (0.0%)	
Bachelor	28 (38.9%)	32 (44.4%)	9 (12.5%)	3 (4.2%)	
Post-graduate	1 (12.5%)	4 (50.0%)	0 (0.0%)	3 (37.5%)	
	Q8 Post Costs 8. We are concerned about post-implantation expenses, such as device maintenance and repair, which present a financial challenge for us.				
Mothers' Education	Strongly Disagree	Disagree	Agree	Strongly Agree	Chi Square (Association)
N/A	0 (0.0%)	0 (0.0%)	1 (20.0%)	4 (80.0%)	26.92**
Elementary	0 (0.0%)	2 (18.2%)	3 (27.3%)	6 (54.5%)	
High School	2 (7.4%)	2 (7.4%)	9 (33.3%)	14 (51.9%)	
Bachelor	2 (2.8%)	4 (5.6%)	29 (40.3%)	37 (51.4%)	
Post-graduate	3 (37.5%)	0 (0.0%)	0 (0.0%)	5 (62.5%)	

* Statistically significant (*p* < 0.05).

** Very statistically significant (*p* *<* 0.01).

### Descriptive analysis of GCBI questionnaire

The chi-square goodness-of-fit test revealed that the distribution significantly differed from an equal distribution (*p* < .001), indicating strong trends in participants' responses. In the functional and life quality domains, 88.1% of participants reported that the overall life impact was better post-implantation, with similarly high improvements noted in task ability (69.3%) and behavior change (75.2%). Developmental outcomes showed mixed but positive trends, while 60.4% reported improved developmental growth and 31.7% noted no change. Daily energy showed a similar pattern, with 62.4% reporting improvement and 30.7% reporting no change. No change was noted in sleep quality, with 60.4% of participants reporting “no change.” Cognitive and academic areas, such as mental focus (47.5%) and learning ability (72.3%), showed significant improvements, with the majority reporting enhanced performance.

In the social-emotional domain, significant improvements were observed in family integration (73.3%), friendship time (72.3%), and self-confidence (66.3%). On the other hand, social shyness and emotional control showed more moderate distributions between “much better” and “no change” (43.6%; 39.6%), respectively. Happiness (71.3%) and self-image (60.4%) also demonstrated notably positive outcomes, with the majority reporting significant improvements in these areas. While self-care showed notable improvement (62.4%), the perceived changes in health-related indicators were less consistent. In the medical domain, illness frequency (60.4%), doctor visits (56.4%), and medication use (73.3%) had the highest percentages of “no change.” Table 8 for a Descriptive Analysis of GCBI Questionnaire.

**Table 8:** Descriptive analysis of GCBI questionnaire.

Item/Question	Much Worse	Slightly Worse	No Change	Slightly Better	Much Better	X^2^ GoF
**Q1_Life_Impact:** 1.Has your child's operation made his/her overall life better or worse?	0 (0.0%)	0 (0.0%)	2 (2.0%)	10 (9.9%)	**89** **(88.1%)**	137.37***
**Q2_Task_Ability:** 2. Has your child's operation affected the things he/she does?	0 (0.0%)	2 (2.0%)	15 (14.9%)	14 (13.9%)	**70** **(69.3%)**	109.89***
**Q3_Behavior_Change:** 3. Has your child's operation made his/her behavior better or worse?	0 (0.0%)	4 (4.0%)	5 (5.0%)	16 (15.8%)	**76** **(75.2%)**	139.52***
**Q4_Development_Growth:** 4. Has your child's operation his/her progress and development?	0 (0.0%)	0 (0.0%)	32 (31.7%)	8 (7.9%)	**61** **(60.4%)**	41.84***
**Q5_Daily_Energy:** 5. Has your child's operation affected how lively he/she is during the day?	0 (0.0%)	1 (1.0%)	31 (30.7%)	6 (5.9%)	**63** **(62.4%)**	95.71***
**Q6_Sleep_Quality:** 6. Has your child's operation affected how well he/she sleeps at night?	1 (1.0%)	3 (3.0%)	**61** **(60.4%)**	5 (5.0%)	31 (30.7%)	132.52***
**Q7_Eating_Enjoyment:** 7. Has your child's operation affected his/her enjoyment of food?	2 (2.0%)	2 (2.0%)	**48** **(47.5%)**	6 (5.9%)	43 (42.6%)	106.77tn
**Q8_Self_Awareness:** 8. Has your child's operation affected how self-conscious he/she is with other people?	0 (0.0%)	3 (3.0%)	**48** **(47.5%)**	19 (18.8%)	12 (11.9%)	97.14***
**Q9_Family_Integration** 9. Has your child's operation affected how well he/she gets on with the rest of the family?	0 (0.0%)	1 (1.0%)	18 (17.8%)	8 (7.9%)	**74** **(73.3%)**	131.28***
**Q10_Friendship_Time:** 10. Has your child's operation affected his/her ability to spend time and have fun with friends?	0 (0.0%)	1 (1.0%)	17 (16.8%)	10 (9.9%)	**73** **(72.3%)**	125.50***
**Q11_Social_Shyness:** 11. Has your child's operation affected how embarrassed he/she is with other people?	1 (1.0%)	6 (5.9%)	**44** **(43.6%)**	10 (9.9%)	40 (39.6%)	80.83***
**Q12_Mental_Focus:** 12. Has your child's operation affected how easily distracted he/she been?	1 (1.0%)	2 (2.0%)	40 (39.6%)	10 (9.9%)	**48** **(47.5%)**	97.47***
**Q13_Learning_Ability:** 13. Has your child's operation affected his/her learning?	0 (0.0%)	2 (2.0%)	15 (14.9%)	11 (10.9%)	**73** **(72.3%)**	123.91***
**Q14_Absence_Frequency:** 14. Has your child's operation affected the amount of time he/she has had to be off nursery, playground, or school?	1 (1.0%)	4 (4.0%)	**47** **(46.5%)**	4 (4.0%)	45 (44.6%)	110.24***
**Q15_Task_Focus:** 15. Has your operation affected his/her ability to concentrate on a task?	0 (0.0%)	2 (2.0%)	30 (29.7%)	6 (5.9%)	**63** **(62.4%)**	93.42***
**Q16_Emotional_Control:** 16. Has your child's operation affected affected how frustrated and irritable he/she is?	2 (2.0%)	8 (7.9%)	32 (31.7%)	13 (12.9%)	**46** **(45.5%)**	66.18***
**Q17_Self_Image:** 17. Has your child's operation affected affected how he/she feels about himself/herself?	0 (0.0%)	2 (2.0%)	21 (20.8%)	17 (16.8%)	**61** **(60.4%)**	75.44***
**Q18_Happiness:** 18. Has your child's operation affected affected how happy and content he/she is?	0 (0.0%)	2 (2.0%)	18 (17.8%)	9 (8.9%)	**72** **(71.3%)**	120.51***
**Q19_Confidence:** 19. Has your child's operation affected affected his/her confidence?	0 (0.0%)	3 (3.0%)	22 (21.8%)	9 (8.9%)	**67** **(62.4%)**	99.52***
**Q20_Self_Care:** 20. Has your child's operation affected affected his/her ability to care for himself/herself as well as you think they should, such as washing, dressing, and using the toilet?	1 (1.0%)	0 (0.0%)	25 (24.8%)	12 (11.9%)	**63** **(62.4%)**	86.68***
**Q21_Recreation_Sport:** 21. Has your child's operation affected his/her ability to enjoy leisure activities such as swimming and sports, and general play?	1 (1.0%)	13 (12.9%)	27 (26.7%)	12 (11.9%)	**48** **(47.5%)**	64.69***
**Q22_Illness_Frequency:** 22. Has your child's operation affected how prone he/she is to catch colds or infection?	1 (1.0%)	10 (9.9%)	**61** **(60.4%)**	8 (7.9%)	21 (20.8%)	113.21***
**Q23_Doctor_Visits:** 23. Has your child's operation affected how often he/she needs to visit a doctor?	2 (2.0%)	8 (7.9%)	**57** **(56.4%)**	7 (6.9%)	27 (26.7%)	101.72***
**Q24_Medication_Use:** 24. Has your child's operation affected how much medication he/she has needed to take?	1 (1.0%)	3 (3.0%)	**74** **(73.3%)**	2 (2.0%)	21 (20.8%)	192.61***

**Bold** values represent the most frequently selected option of questions with statistically significant responses (*p* < 0.05 or lower).

*** Extremely statistically significant (*p* < .001).

#### Association between variables of GCBI questionnaire

The chi-square test of independence revealed statistically significant associations across multiple variables (Table 9). For instance, day care or school attendance was significantly associated with perceived changes in friendship time [*χ*^2^(3, *n* = 101) = 8.02, *p* = .046], with a moderate effect size (Cramér's V = .282). Children who attended daycare/school were more likely to report having “much better” friendship time than those who did not attend. Similarly, age at cochlear implantation was significantly associated with parent-reported changes in children's daily energy [*χ*^2^(9, *n* = 101) = 19.38, *p* = .022], which was further supported by Fisher's exact test (*p* = .019). Children implanted before the age of 2 years were more likely to exhibit noticeable improvements in daily energy than those implanted after the age of 6 years.

**Table 9:** Association between variables of GCBI questionnaire.

	Q10 Friendship Time				
Daycare/School Attendance	Slightly worse	No change	Slightly better	Much better	Chi-Square (Association)
No	0 (0.0%)	10 (32.3%)	3 (9.7%)	18 (58.1%)	8.02*
Yes	1 (1.4%)	7 (10%)	7 (10%)	55 (78.6%)	
	Q5 Perceived Change in Daily Energy				
Age of CI Implantation	Slightly worse	No change	Slightly better	Much better	Chi Square (Association)
Less than two	1 (3.8%)	6 (23.1%)	0 (0.0%)	19 (73.1%)	19.38*
Two to three	0 (0.0%)	15 (31.3%)	1 (2.1%)	32 (66.7%)	
Four to five	0 (0.0%)	5 (31.3%)	2 (12.5%)	9 (56.3%)	
Six or more	0 (0.0%)	5 (45.5%)	3 (27.3%)	3 (27.3%)	
	Q1 Perceived life Impact				
Father's Occupation	Slightly worse	No change	Slightly better	Much better	Chi Square (Association)
Unemployed	1 (33.3%)	1 (33.3%)	1 (33.3%)	3 (33.3%)	23.78**
Education	0 (0.0%)	3 (14.3%)	18 (85.7%)	21 (100.0%)	
Business	0 (0.0%)	0 (0.0%)	10 (100.0%)	10 (100.0%)	
Health Care	0 (0.0%)	0 (0.0%)	6 (100.0%)	6 (100.0%)	
Office Work/Management	1 (4.0%)	4 (16.0%)	20 (80.0%)	25 (100.0%)	
Other	0 (0.0%)	2 (5.6%)	34 (94.4%)	36 (100.0%)	

* Statistically significant (*p* < .05)

** Very statistically significant (*p* *<* .01).

*** Extremely statistically significant (*p* < .001).

A significant relationship was also found between fathers' occupation and perceived life impact [*χ*^2^(10, *n* = 101) = 23.78, *p* = .008], with a moderate effect size (Cramér's V = .343); however, Fisher's exact test was borderline (*p* = .068). Working fathers reported “much better” improvement in their child's life impact whereas, non-working fathers mostly reported that “no change” was observed. Additionally, there was a statistically significant [*χ*^2^(15, *n* = 101) = 41.36, *p* < .001] and a moderately strong relationship (Cramér's V = .369) between mothers' occupation and changes in children's daily energy levels, which was confirmed by Fisher's exact test (*p* = .043). Improvements in children's energy levels are more commonly associated with unemployed mothers.

## Discussion

This study aimed to explore parents' perceptions of barriers to pediatric cochlear implantation and its perceived benefits for their children. One of the barriers to timely access to CI is the early detection of hearing loss (15, 19). Despite the mandatory NBHS program in Saudi Arabia, this study revealed a high prevalence of children who had not undergone NBHS (42.3%), which likely contributed to delayed identification of hearing loss. This delay is reflected in over half of the children in the study being diagnosed at or after nine months of age (56.1%), which exceeds the critical 1-month window for early identification recommended by the 1-3-6 guidelines (25). In more recent years, Alothman et al. (26) reported a national NBHS coverage rate of 92.6%, showing promising progress.

The demographic and clinical summary of this study revealed that 70.7% of parents reported consanguineous marriage, although the specific degrees of familial relations were not assessed. The high prevalence of bilateral hearing loss, along with the substantial rate of consanguinity observed, may suggest an underlying genetic component (27, 28). This interpretation is supported by a systematic review by Aljabri et al. (27), who reported consanguinity rates ranging from 21.1% to 80.8%, which are significantly associated with an increased risk of congenital hearing loss in Saudi Arabia.

Clinical findings from the current sample also indicated that among CI recipients, 62.5% of children were implanted bilaterally, while 37.5% of children received unilateral implantation. This distinction is clinically relevant, as Eskridge et al. (29) found that children with bilateral CIs demonstrated significantly better receptive and expressive language outcomes at age five than those with unilateral implants. Regarding the age at implantation, the majority (46.2%) of the children in the study underwent the procedure between the ages of 2 and 3 years, while only (26.9%) received implants before the age of 2 years. These findings fall short of the optimal window recommended by Dettman et al. (7), who emphasized that implantation before 12 months of age supports age-appropriate language acquisition, improved speech production, and enhanced auditory outcomes.

Parental barriers to the pediatric cochlear implantation questionnaire revealed that the majority (80.5%) of parents disagreed that surgical guidance, including clear information and guidance about the rehabilitation plan and expected outcomes, was a barrier. Additionally, there is a strong association between barriers to surgical guidance and parents' educational level. Notably, mothers with postgraduate education were significantly more likely to express strong concerns about the adequacy of surgical guidance provided prior to implantation. This suggests that higher educational attainment is associated with increased expectations for detailed medical communication and transparency in decision-making. A similar pattern was observed in Aelbrecht et al.'s (39) study on patient–physician communication, which found that participants with lower levels of education tended to prioritize emotional and relational aspects of communication, while those with middle to higher education levels focused more on task-oriented, problem-solving aspects such as the clarity of medical information conveyed.

The results revealed that a considerable proportion of parents (45.5%) expressed concerns regarding the surgical procedure, including potential risks and complications related to the surgery. This aligns with the findings of Dev et al. (13) and Ibrahim et al. (18), both of which reported that parents often experience anxiety related to surgery and its possible complications when considering cochlear implantation. In addition, comprehensive pre-implantation assessments, such as MRI, play a crucial role in identifying anatomical or medical concerns in advance, enabling surgeons to tailor their surgical approach, thereby reducing the likelihood of intraoperative or postoperative complications (30).

In addition to surgical concerns, a significant number of parents were concerned about the potential difficulties accessing rehabilitation services, including device programming and technical support as well as speech therapy, due to the limited availability of centers in their area. Preoperative home-based therapy during the hearing aid trial typically involves parent-guided auditory stimulation; however, postoperative CI rehabilitation is more structured and requires regular clinical follow-up, mapping sessions, and professional speech-language intervention. In areas where access to specialized centers is limited, home-based therapy may serve as a complementary strategy but does not replace formal post-implant rehabilitation services. This may be attributed to regional disparities in the distribution of rehabilitation centers across Saudi Arabia, as noted by Elbeltagy et al. (31), who reported that CI rehabilitation services are more commonly offered in Riyadh than in other regions. Notably, 54.5% of our participants were from the Makkah region, which suggests a shortage of rehabilitation facilities in that area, contributing to the reported access challenges. Difficulty in accessing rehabilitation services due to regional disparities in service availability remains a common barrier to consistent post-implantation care, not only in Saudi Arabia but also globally (20). Results of the current study revealed that access to rehabilitation was also associated with family income. For example, as expected, higher-income families (≥20,000 SAR/month) were less likely to report having concerns about accessing rehabilitation services compared to lower-income families, likely due to their ability to afford private services, transportation, or relocation if necessary.

Other burdens identified in the study included post-implantation expenses, with nearly half of the parents reporting that the cost of device maintenance (49.6%) and ongoing rehabilitation sessions (48.8%) posed substantial challenges. Moreover, a clear association was observed between family income and postsurgical costs, such as device maintenance and repair. These findings are consistent with those reported by Ibrahim et al. (18). Additionally, Khamayseh (32) found that more than half of parents expressed fear related to the post-implantation device and spare parts.

A notable proportion of parents reported concerns about the limitations their children might face, particularly regarding participating in sports activities (41.5%) and undergoing MRI (38.2%). However, recent evidence indicates that advancements in CI technology have significantly mitigated MRI compatibility concerns, particularly at 1.5 Tesla, reducing risks and enhancing safety for CI recipients (40). Similarly, concerns about sports participation have been addressed through protective covers that allow for the safe use of CIs during water exposure and humid conditions (33). These innovations have expanded opportunities for engagement in swimming and other physical activities, addressing prior limitations related to device vulnerability.

To complement the analysis of parental barriers, this study also examined parent-reported outcomes regarding their children's QoL following cochlear implantation, as measured by the GCBI. The GCBI assesses perceived changes across the functional, developmental, cognitive, social-emotional, and medical domains. The following discussion highlights the most significant perceived benefits of the intervention, focusing on aspects within each domain where improvements were reported by 70% or more of the parents.‏ Generally, the current study found that participants perceived a positive outcome after implantation, with 88.1% reporting improved overall life impact. This finding aligns with previous research indicating that cochlear implantation enhances QoL, as reported by parents and caregivers (18, 21). In addition, an association was found between fathers’ employment status and their perceptions of their child's life impact following implantation, with working fathers reporting significant improvements in their child's overall QoL.

Within the functional domain of the GCBI, 75.2% of parents reported noticeable improvements in their children's behavior following cochlear implantation. This aligns with the findings of Sari et al. (34), who reported that children exhibited fewer post-implantation behavioral issues, particularly in the areas of aggression, social interaction, and attention regulation. Such outcomes underscore the potential of CIs to improve self-regulation and behavioral functioning. Additionally, Saki et al. (35) demonstrated a strong inverse relationship between speech intelligibility and aggression levels, reinforcing that enhanced communication abilities may contribute to more positive behavioral outcomes.

Our study found that 72.3% of parents of children with CIs reported significant improvements in their children's learning ability following implantation, which is consistent with prior research demonstrating the educational benefits of CIs. Cejas et al. (36) found that children with CIs, particularly those implanted before 18 months of age, showed stronger academic performance, language development, and QoL compared to peers with similar degrees of hearing loss who did not receive implants. These findings suggest that early auditory access through the CI supports foundational language skills that are critical for academic success. Similarly, Alsari (37) reported substantial improvements in speech and language outcomes among prelingually deafened Arabic-speaking children following CI, reinforcing the global relevance of these benefits. Together, these findings indicate that CI not only improves auditory and speech outcomes, but also meaningfully enhances educational performance, as supported by both clinical assessments and parent-reported experiences.

Finally, significant improvements in the social-emotional domain following cochlear implantation were evident, with the majority of parents reporting notable positive outcomes in family integration (73.3%), friendship time (72.3%), and overall happiness (71.3%). These findings indicate that the impact of cochlear implantation extends beyond auditory benefits, supporting children's emotional and social development. According to Shah et al. (38), enhancement in family integration likely reflects better communication within the household, allowing the child to participate more fully in daily interactions and strengthen familial bonds. Similarly, the rise in reported happiness may be attributed to increased independence and self-expression, which positively influences children's emotional well-being. Improvements in friendship time suggest that children with CIs are more capable of engaging in peer interactions and social activities, thereby fostering the development of critical social skills (38).

### Limitations

This study had several limitations that should be considered. The sample size fell short of the target size, which may have limited the statistical power and generalizability of the findings. The use of convenience sampling from CI clinics in Jeddah and online platforms may have introduced a selection bias, and the sample may not reflect the broader Saudi population. There was also an imbalance in participant groups, with relatively few parents of CI candidates compared to recipients, which limited the representation of preimplantation parental perspectives. Most respondents were from the Makkah region, which reduced the sample's geographic diversity. Additionally, the low response rates for some items may have led to inflated percentage-based interpretations. Future studies should include more representative samples and objective outcome measure to complement the parent-reported outcomes and minimize potential recall bias, especially for parents of children implanted several years earlier.

## Conclusion

This study examined parental perspectives on the barriers and perceived benefits of pediatric cochlear implantation in Saudi Arabia. The most perceived barriers included financial concerns, limited access to rehabilitation services, and specific postsurgical limitations. The findings indicate that while several challenges persist, cochlear implantation is largely associated with positive outcomes, particularly in the functional, cognitive/academic, and social-emotional domains.

## Data Availability

The raw data supporting the conclusions of this article will be made available by the authors, without undue reservation.
